# In the beginning was the word: How terminology drives our understanding of endosymbiotic organelles

**DOI:** 10.15698/mic2019.02.669

**Published:** 2019-01-21

**Authors:** Miroslav Oborník

**Affiliations:** 1 Biology Centre CAS, Institute of Parasitology, České Budějovice, Czech Republic.; 2 University of South Bohemia, Faculty of Science, České Budějovice, Czech Republic.

**Keywords:** bacterium, eukaryote, organelle, evolution, microbiome, endosymbiosis, domestication

## Abstract

The names we give objects of research, to some extent, predispose our ways of thinking about them. Misclassifications of Oomycota, Microsporidia, Myxosporidia, and Helicosporidia have obviously affected not only their formal taxonomic names, but also the methods and approaches with which they have been investigated. Therefore, it is important to name biological entities with accurate terms in order to avoid discrepancies in researching them. The endosymbiotic origin of mitochondria and plastids is now the most accepted scenario for their evolution. Since it is apparent that there is no natural definitive border between bacteria and semiautonomous organelles, I propose that mitochondria and plastids should be called bacteria and classified accordingly, in the bacterial classification system. I discuss some consequences of this approach, including: i) the resulting “changes” in the abundances of bacteria, ii) the definitions of terms like microbiome or multicellularity, and iii) the concept of endosymbiotic domestication.

## INTRODUCTION

Terminology is the basis of science: the words we use for describing the world around us substantially affect the way we think about objects of research. Taxonomy is a typical example of fundamental and frequently used scientific terminology. In my opinion a taxonomic classification, apart from its direct relevance to phylogeny and evolution, is also an important determinant for the choice of field-specific scientific methods and approaches for investigations of a particular organism. There are several groups of organisms that have relatively recently been shown to have fundamentally different phylogenetic positions than their traditional classifications, some of which had been used for centuries. Microsporidia is one example I would like to mention here. These intracellular eukaryotic parasites were first classified in 1857 as schizomycete fungi, an artificially composed group (in today's perspective) containing bacteria in addition to fungi and yeasts. Microsporidia were reclassified to Sporozoa in 1882, specifically to Cnidosporidia, another conglomerate of then unclassifiable groups of intracellular parasitic organisms such as Microsporidia, Myxosporidia, Actinosporidia, and Helicosporidia [1]. An extreme reduction of eukaryotic cellular structures in microsporidia, mainly the absence of a visible mitochondrion, led to the suggestion that they had diverged from other eukaryotes before these structures had evolved [2]. Moreover, early molecular phylogenetic methods supported the very ancient eukaryotic origin of microsporidia [3-6]. However, more sophisticated phylogenetic analyses together with an increase in analyzable molecular data placed microsporidia once again with fungi [7, 8]. The fields of microbiology, mycology and protistology, use different terminologies and approaches, and definitely have different “evolutionary histories”, not least because they were developed by different leading personalities. The fact that fungi have been studied by protistologists for more than 100 years has various negative consequences, including the impending invalidation of all microsporidian species descriptions [1, 9]. Because protistological ultrastructural and morphological terminology is not compatible with mycological terminology, we are in a situation where we have no clue about the origins of intracellular structures in microsporidia and we do not know the homologies to corresponding structures in fungi. To some extent, this also applies to their physiology and ecology. Similar levels of methodological and taxonomic confusion can also be found in other previously misclassified groups of organisms such as Myxosporidia (animals studied by protistologists), Oomycota (stramenopiles studied by mycologists), or Helicosporidia (green algae studied by protistologists). The specific scientific terminology employed by scientists trained in a particular field obviously determines the methods, approaches, and scientific concepts applied to certain groups of organisms. Here, I identify and discuss the bias arising from taxonomic separation of semiautonomous organelles – mitochondria and plastids – from the bacterial classification system, although they are clearly of bacterial origin and thus, in my opinion and according to the phylogenetic species concept, should be classified and studied as bacteria.

## MITOCHONDRIA AND PLASTIDS SHOULD BE CLASSIFIED AS BACTERIA

It is now widely accepted that eukaryotic organelles – mitochondria and plastids – evolved from a free-living bacterial ancestor in a process called primary endosymbiosis [10-12]. This process includes not only an engulfment of free-living bacteria, but also massive gene losses, a transfer of genes from the bacterial cell to the eukaryotic host nucleus, and the evolution of a mechanism for importing nuclear encoded proteins into the domesticated bacteria mediated by transit peptides or eventually by bipartite targeting sequences in secondary and other complex plastids. But do all these processes negate the bacterial identity of organelles? Attempts to treat and name semiautonomous organelles as bacteria are not brand new: for example, this was proposed in 2011 by British microbiologist Mark J. Pallen [13], who summarized and discussed most of the positives and negatives of such an approach. There are many arguments raised against microbial classification of bacteria-derived organelles, mostly regarding their high genetic and metabolic integration to the host cell and the mosaic evolutionary origin of their proteomes. However, works of various scientists [14-16] have shown that symbiotic bacteria can also be deeply genetically integrated with the host organism: they can use proteins that are not encoded in their DNA, they are definitely not able to live without the host, and they can be essential for the functioning of the host cell. All these qualities have been attributed to organelles. Frankly speaking, there is actually no well-defined border between symbiotic bacteria and semiautonomous organelles [14-17]. It has been proposed that the transfer of a gene from a symbiont to the host nucleus followed by import of the corresponding protein from the cytoplasm into the symbiont should define the transition to an organelle [18], but is one such gene enough? Do human patients lose their human identity when they cannot express one particular gene, and need to have the protein externally supplied? It is obvious that plastids and mitochondria are called organelles simply because their bacterial origin was not widely accepted until the second half of the twentieth century [19]. Their naming, which does not respect their bacterial identity, is therefore based solely on tradition rather than scientific usefulness. It should be mentioned here that the naming of mitochondria and plastids as bacteria would not be an easy job, as there are many eukaryotic species and each of them may contain a different species of symbiotic α-proteobacterium (i.e. mitochondrion). This problem could easily be solved by adding a prefix to the name of the eukaryotic host: for example, the mitochondrion of *Vitrella brassicaformis* would be “*Mitovitrella brassicaformis”*. However, I must admit that a much greater formal obstacle – the rules of bacterial classification - prevents such naming; all the organelles should be named as Candidatus, similar to symbiotic bacteria, because we cannot cultivate them. On the other hand, this rule may be too strict, as it allows us to classify only a minority of bacterial species: it is estimated that about 85-99% of bacteria and archaea cannot be cultivated in the lab [20].

The fact that organelles are not classified as bacteria has several unfortunate consequences. Many (maybe most) people working with mitochondria and plastids do not even think about them as bacteria, but that is what they are: bacteria that just happen to be living in a very specific environment, inside the eukaryotic cell. They do not apply bacteriological methods and approaches to mitochondria and plastids at all. Therefore, I think that it is time we finally call organelles by their appropriate and correct names – bacteria – to thereby avoid the above-mentioned biases in the investigations of misclassified organisms. That said, what would be the effect of calling and treating mitochondria and plastids as bacteria?

## THE WORLD BECOMES MORE BACTERIAL

It is obvious that inclusion of mitochondria and plastids into the framework of bacterial taxonomy would have at least one remarkable effect. The abundance of bacteria in all environments would substantially increase. Also, the total numbers of bacteria estimated to live on earth would have to change. When we take into account the presence of (usually) multiple mitochondria in every eukaryotic cell, the numbers of bacteria of each particular group would climb. As an example, let's look at humans through this new prokaryotic lens. Human cells contain between 0 (red blood cells) and 2,000 (liver cells) mitochondria, depending on the particular tissue. The estimated number of cells in the human body is 3×10^13^ [21]. Even if 100 mitochondria per cell is assumed (which is certainly underestimated), a single human body would contain at least 3x10^15^ mitochondria. The entire human population (approximately 7x10^9^) would thus increase the total number of α-proteobacteria on earth by 2x10^25^. As the diversity of eukaryotes is large, consisting, of course, not only of humans or mammals, the final numbers of mitochondria would be incredibly huge. When plastid endosymbioses are taken into account, the numbers of cyanobacteria would also dramatically increase. Actually, in contrast to mitochondria, we can even see by the naked eye cyanobacteria in plants: all the green color we see around us, in plants and algae, are domesticated cyanobacteria because only they contain chlorophyll. Furthermore, primary production by eukaryotes (including all the rain forests and cultured plants, as well as eukaryotic phytoplankton such as chlorophytes, rhodophytes, glaucophytes, diatoms, chrysophytes, haptophytes, dinoflagellates, and many others) is much larger than that of phototrophic bacteria. Rough estimations indicate that free-living phototrophic bacteria are responsible for about 20-40% of global primary production, while 60-80% of organic carbon is fixed by eukaryotes [22-25]. If the eukaryotic contribution was attributed to the actual producers – cyanobacteria living inside eukaryotic cells – primary production would be a process performed exclusively by bacteria.

What is even more interesting is the fact that primary endosymbioses leading to the presence of what we call “organelles” are quite rare, apparently having happened only twice (once for mitochondria, once for plastids; a second cyanobacterial primary endosymbiosis in cercozoans was relatively recent [26] and resulted in low abundance and diversity and is not considered here). This means that only a single species of α-proteobacteria and a single species of cyanobacteria were able to survive inside a eukaryotic cell and are thus the foundation of eukaryotic diversity. The endosymbiotic strategy along with domestication enabled unprecedented distribution of mitochondria and plastids throughout eukaryotic diversity and throughout global habitats, originating from just those two ancestral bacterial species.

I must admit that even I have a strict border for determining when a symbiotic bacteria has lost its bacterial identity: when the bacterial genome is lost – as seen in some anaerobic mitochondria-derived organelles (mitosomes) [27] and rarely, in non-photosynthetic plastids such as the plastid of colorless green alga in genus *Polytomella* [28] – a symbiotic bacterium has lost the last traces of its autonomy and has become a true organelle.

## DOMESTICATION OR INVASION?

You may have noted that I am talking here about “domestication” of free-living bacteria by a eukaryotic host. At least the acquisition of cyanobacteria by primary endosymbiosis suspiciously resembles the domestication of animals and plants by human beings. Humans killed and ate wild animals as their prey at initial stages of the mutual relationship. Subsequently, the “future host” (humans) found out that some species can live in close proximity without being stressed, with all the additional benefits inaccessible before the domestication event: one can hardly milk wild buffalo. It is noteworthy that only a limited number of animals could be domesticated. Domestication also leads to a mutual dependence of the domestication partners: we would obviously pay a high price for losing our domesticated plants and animals, just as they would. Of course, for a human individual, it is not necessarily as life threatening as the loss of mitochondria would be for a eukaryote (but even this has happened [29]); however, on a population or even on a species level, domestication is kind of essential to our lives. Domestication is evolutionarily advantageous for both partners: the domesticated species would never have reached such high abundances without human support and humans would never have left the hunter and gatherer lifestyle with the corresponding low human population density. Endosymbiotic relationships result in similar advantages to both partners.

But truthfully, all this is purely “the eukaryotic view”. Endosymbioses leading to organelle evolution are usually understood as the engulfment of a bacterium (or an alga in complex endosymbioses), originally as food [12], which has somehow survived in the predator cell without being digested, in line with the domestication hypothesis mentioned above. This scenario supposes that the eukaryotic partner is active in this process: it hunts the prey and later domesticates it. However, although the latest analysis shows that the ancestor of mitochondria was a rather deeply branching α-proteobacteria [30], it has also been speculated that the bacterial ancestor of mitochondria was actually an intracellular parasite [31], similar to the related Rickettsiales. Parasitic ancestry of mitochondria would substantially change the beat, with an active parasitic invasion replacing a passive engulfment of food. It has even been stated that most endosymbiotic bacteria in the evolutionary history of eukaryotic cells were originally pathogenic [32]. Although it is difficult to imagine a phototrophic bacteria being a parasite (in the case of a cyanobacterium as ancestor of plastids), a somehow “intentional” invasion into the eukaryotic host cell could be understood as a great strategy for colonizing new environments and utilizing the advanced cellular equipment of the host cell in order to increase bacterial population and diversity. Moreover, it was recently shown that the phototrophic marine alga *Chromera velia*, which was hypothesized to live as a coral symbiont [33], is likely a facultative parasite of coral larvae [34], so these two trophic modes can, in principle, coexist. Even so, a phagotrophic initiation of plastid acquisition does not weaken the parasitic hypothesis because uptake as food is one of the most common infection mechanisms exploited by parasites: even humans are mostly invaded by food- or waterborne parasites. The ancestor of Archaeplastida could have been regularly eating bacteria, but on one occasion it simply engulfed the cyanobacterium and was colonized by it. The possible pathogenic character of the cyanobacterial ancestor of plastids is also supported by the similarity of plastid transit peptides and α-helical structures on antimicrobial peptides [35].

Thus, the question arises: which partner of the two is actually the domesticated one? It seems difficult for us to accept that a bacterium represents an active partner in the symbiotic relationship and thereby might determine the purpose and the fate of humans and other eukaryotes; yet, from the “bacterial point of view”, humans (and other eukaryotes) would just be very sophisticated motile incubators for bacteria. And in fact, bacteria are our drivers – mitochondria generate most of the energy for our cells, allowing us to function in all the ways that we do. Since we are eukaryotes, it is difficult to admit that some “primitive” prokaryotic cell enslaved an ancestor of eukaryotes and that we are only here to provide a safe space for our bacterial lords; however, this psychological problem does not change the fact that the cells of our ancient single-celled ancestors some billions of years ago were invaded by bacteria. And here, we have returned to my point of changing the terminology: choosing and adopting the “eukaryotic” or the “bacterial” view of endosymbiosis predisposes our way of thinking about it.

There are many different bacteria living inside eukaryotic cells [36]. As far as I know, many of them are not covered by the endomembrane envelope, just as mitochondria and primary plastids. It is apparent that the range of hosts that were able to accept the ancestors of primary plastids and mitochondria is extremely limited because both primary endosymbioses are singular events believed to have happened only once in a billion years. To encase bacteria by an additional membrane of eukaryotic origin appears to be a highly successful strategy for invading a broader range of hosts. In the evolution of phototrophic organelles, much greater numbers of secondary and higher order endosymbioses [12, 37-39] than primary plastid endosymbiotic events [10-12] have occurred in the evolution of eukaryotes, suggesting that it is much easier for a eukaryotic host to accept eukaryotic (algal) rather than prokaryotic phototrophic symbionts. Interestingly, secondary endosymbiotic strategies appear to occur exclusively in aquatic organisms, while primary eukaryotic phototrophs were also successful in terrestrial habitats. The case of algae with complex plastids also illustrates that phototrophy may not be necessarily as beneficial for the host cell because many algae with secondary plastids have lost the ability to photosynthesize and have switched to hetero-osmotrophy, predation, or parasitism [10, 11]. Therefore, the trophic motivation of the host cell to capture the phototrophic symbiont may not be as strong as one would expect. At the same time, most non-photosynthetic algae still contain the relic plastid, which retains essential functions for the host. Such mutual dependence of the host and symbiont again resembles, to some extent, the relationship between humans and domesticated species.

## INTRACELLULAR AND EXTRACELLULAR MICROBIOMES

The collection of bacteria accompanying many eukaryotic species is called the microbiome [41]. Since mitochondria are not understood and studied as bacteria, they are consequently not included in the current view of the eukaryotic microbiome, although they inhabit the human (or any other eukaryote) body. The number of bacteria constituting the human microbiome was estimated to reach 3.8x10^13^ [16], which is comparable to the number of eukaryotic cells composing the human body (3.0x10^13^) [16]; a fact that is usually surprising to non-biologists. If mitochondria are counted as bacteria and consequently incorporated into the eukaryotic (human) microbiome, the entity we call the eukaryotic cell would become a minor component of our bodies, at least by cell counts. Mitochondria and other intracellularly symbiotic bacteria may thus constitute what should be called “the intracellular microbiome”.

Analogously, the presented proposal also affects the concept of multicellularity: it is actually difficult to talk about single-celled eukaryotes when the vast majority of them contain mitochondria, which, according to my suggestion, are cells living inside of them. In fact, this means that all eukaryotes (with the exception of Monocercomonoides [29]) represent multicellular assemblies. I therefore suggest a distinction between vertical (a eukaryote with its intracellular microbiome) and horizontal (a eukaryote that consists of differentiated cell types) multicellularity. The only single-celled organisms would thus be bacteria and archaea without an intracellular symbiont.

## THE EUKARYOTIC CELL AS AN INTIMATELLY INTEGRATED MICROBIAL COMMUNITY

The proposed concept could also change our view on human beings, which would become much more connected to our biological nature. The understanding that bacteria are our intimate friends, not only living on our skin and in our mouths and guts, but also inside our very cells along with the knowledge that our evolution is inseparably linked to our intracellular bacterial symbionts, will hopefully be refreshing in our postindustrial world full of antibacterial soaps. The eukaryotic cell should be understood more as a kind of highly sophisticated and extremely closely integrated microbial community than as a strictly isolated and independent biological entity. In this way, when naming the microbial components of eukaryotic cells, we may be more likely to see the features they have in common with free living bacterial ancestors, allowing us to better understand their nature and function rather than looking for differences just to justify our use of the traditional terminology. A microbial community is defined as a “multi-species assemblage in which organisms live together in a continuous environment and interact with each other”. In the past, the microbial community was even seen as a “supra-organism” [42]. It has recently been shown [43] that genetic processes such as gene loss and consequential genome reduction in entities named eukaryotic organelles (mitochondria and plastids) also happens in components of microbial communities, leading to a conglomerate of microbial species with diverged metabolic abilities. Such gene loss in “free-living” organisms results in a network of participants with different levels of genome reduction and different repertoires of lost and retained metabolic pathways, making all involved microbes strictly dependent on their cohabitants [43]. In spite of the similar genetic processes occurring in microbial communities and eukaryotic organelles, no one would doubt the bacterial nature and identity of bacterial components of complex microbial communities. I am convinced that we should try to study the eukaryotic cell as a highly integrated and to some extent dynamic microbial community in order to fully understand the origins and complexity of cellular processes. Knowing the former autonomy of individual biological components, we can better understand their function in a complex system: the eukaryotic cell.

We can always find some exception to any strict definition of an organelle. I am afraid that I will not persuade scientists to change the classification of organelles to the bacterial one, even if I am convinced that such a change would be beneficial for science. I must concede that the history of science deeply influences scientific terminology [44]. So I simply want to show here that we can think about life forms differently, besides the concepts commonly accepted and taught. Nevertheless, I am afraid that the concept of organelles, as it is proposed by Ansgar Gruber [45], which in effect would classify many symbiotic bacteria (not only mitochondria and plastids) as organelles, would be equally unacceptable for the scientific public as my proposal for naming organelles as bacteria. Neither proposal can satisfy everyone.

## RESPONSE TO ANSGAR GRUBER

### Genetic and metabolic integration

Genetic integration, evidenced by the import of a nuclear encoded protein into the symbiont, has been widely accepted as the defining feature that makes an organelle [18]. According to this concept, some symbiotic bacteria should in fact be named as organelles: for example, some bacterial symbionts of insects [14-17] can be genetically integrated to the host by using proteins encoded in the host nucleus for their metabolism. Metabolic integration is obviously very common, not only in organelles [12-17] and microbial communities [42, 43]; actually, the dependence of humans on domesticated species is also metabolic – we use products of animal and plant metabolism to feed our own metabolism. Furthermore, some specific essential metabolic products can be externally supplied to deficient humans: for example, insulin can be produced by animals or even by genetically modified bacteria with the human gene inserted into their genome, and used to supply insulin-deficient humans. A similar situation applies for essential amino acids, vitamins, and other compounds. Metabolic integration is generally the basis of the functioning of any ecosystem, resulting in a network of various producers and consumers. To me, it is not important that the components of these ecosystems are usually not living inside a single cell because they are using metabolic compounds accessible from their environment in principally the same way as do organelles and symbionts. I would personally apply this concept also to multicellular organisms, since individual differentiated cells metabolically depend on each other and behave as components of a microbial community. An extreme example of such (sometimes even “selfish”) behavior can be seen in cancer cells, which become a kind of pathogen, damaging the multicellular body. In some cancer cells, mitochondria can even migrate between the cells like regular bacteria [46, 47]. This results in the medical approach called tumor ecology [48], which applies the above concept in practice. I would note that practical medicine is sometimes (but rarely) ahead of theoretical science. For example, eukaryotic parasites such as the causative agent of malaria, *Plasmodium*, can be treated by antibiotic compounds such as Rifampin; thus, in practice, their organelles are treated as bacteria [49].

A frequent argument for not treating and naming mitochondria and plastids as bacteria is the mosaic evolutionary origin of their proteome. However, bacteria are generally known to be prone to massive horizontal gene transfer (HGT), resulting in an extensive evolutionary mosaicism of their genomes and consequently proteomes [50,51]. Quite frequent HGT was noted particularly in microbial communities where some species can serve as a kind of “gene bank”, holding genes that are then redistributed to the community [51]. Bacterial HGT is also involved in the phenomenon of pathogenicity islands, in which particular regions of prokaryotic genomes, from 10 to 200 kb in length, functioning as virulence factors, are transferred between bacterial species [52]. Also, some single-celled eukaryotes like diatoms contain many bacterial genes in their genomes (and proteomes); for example, the pennate diatom, *Phaeodactylum tricornutum*, has been shown to contain more than 600 bacterial genes [53]. Are these complex algae therefore less eukaryotic? In diatoms, such bacterial HGT-originating genes can be involved in quite important and unique pathways, such as the syntheses of toxins (for example, neurotoxic domoic acid [54]). Similarly, HGT from eukaryotes to bacteria has been shown: for example, *Legionella* contains more than 100 eukaryotic-like proteins and *Wolbachia* shares salivary gland surface proteins with mosquitos (HGT is probably occurring in both directions) [55]. Does this mean that they are not bacteria anymore? Last but not least, nobody would call viruses organelles, although they are likely the most closely genetically host-integrated biological entities we know: they cannot reproduce without the host cell and temperate (latent) viruses can be in a form of DNA fully integrated into the host genome, they are then transmitted to progeny through DNA replication of the host cell. Additionally, their protein envelopes can, in principle, be entirely composed of nuclear encoded proteins [e.g. 56].

### Benefit to the host

Gruber mentioned that a benefit to the host could be a possible criterion for the transformation of an endosymbiont into an organelle [57]. It is rather difficult to talk about a real benefit to the host in the case of organelles, which possess essential metabolic pathways that were present in the ancestor of the host cell before endosymbiont acquisition, but which have since been replaced by the endosymbiont pathway. In my opinion, the beneficial character of pathways, such as the synthesis of iron-sulphur clusters, fatty acids, or isoprenoids, is questionable because they did not bring any benefit to the ancestral symbiont-free cell. The only real benefit is the acquisition of a function or a metabolic pathway that was absent from the host cell before obtaining its symbiont: for example, oxidative phosphorylation or photosynthesis. There is no doubt that oxidative phosphorylation and photosynthesis are beneficial to the host, but at the same time, these pathways are obviously not essential for the host cell because there are numerous non-photosynthetic plastids and anoxic mitochondria without oxidative phosphorylation. In these cases, the organelles have lost their original beneficial function. Therefore, are organelles that lack the original beneficial pathways still organelles?

Likewise, some symbiotic bacteria supply the host cell with essential metabolic products that were not present in the symbiont-free ancestor. For example, endosymbiotic bacteria in trypanosomatids, such as *Herpetomonas roitmani, Crithidia deanei, Crithidia oncopelti*, and *Blastocrithidia culicis*, provide heme and heme precursors for the host cell and the ancestral symbiont-free trypanosomatid was not able to synthesize these essential compounds [58]. Obviously, they are beneficial to the host and should be, according to this concept, named organelles.

### Sexual symbiont integration

The concept of the “sexual integration of an organelle” is the main argument of Ansgar Gruber in this discussion. This concept states that a symbiont becomes an organelle when it has lost host-independent reproduction and speciation. Returning to the parallel of the domestication of animals and plants by humans, control over reproduction and breeding (a kind of artificial speciation) of domesticated species is the first and basic condition for a successful domestication process, not the result of it. In other words, it is a domestication tool rather than a consequence of domestication testifying to the level of integration. Even more importantly, numerous obligatory symbiotic bacteria co-evolve and co-speciate with their hosts, losing their independent reproduction [36, 59] and therefore, according to Gruber's conceptualization, might be considered organelles. It should also be taken into account that plastid and mitochondrial genes do not undergo sexual recombination, although the nuclear encoded genes for proteins targeted to these organelles do; thus, it is difficult to talk about any “sexual” integration of organelles, because nuclear encoded genes that support organellar metabolism are no longer of organellar location and, in many cases, they are not organellar even in their evolutionary origins.

Complex plastids of secondary, tertiary and higher endosymbiotic origins display various levels of integration with the host cell. In addition to fully integrated plastids surrounded by three and four membrane envelopes, in cryptophytes and chlorarachniophytes we can find less integrated plastids with the remnant of an endosymbiont nucleus called a nucleomorph. Likewise, in dinotoms – dinoflagellates with a diatom-derived plastid (endosymbiont) – the photosynthetic symbiont displays very little integration, still containing its mitochondria, nucleus, and the typical diatom plastid. Although the reproduction and division of the diatom endosymbiont is obviously synchronized with the host cell [60], it is very likely that there is no genetic integration because no nuclear encoded proteins are targeted to the symbiont [61]. Such integration is prevented by the single membrane envelope of the symbiont, a likely remnant of the cytoplasmic diatom membrane, which does not allow canonical protein import and therefore results in unprecedented conservation of the endosymbiont [39]. In addition, metabolic integration of the diatom endosymbiont is probably below the standard of other plastids. It has been shown that dinotoms possess two redundant pathways for heme biosynthesis, but both pathways are of plastid origin (aminolevulinate is synthesized from glutamate through the so called C5 pathway), suggesting the presence of two plastids inside the host cell: the remnant of the original peridinin pigmented dinoflagellate plastid, likely in the form of the eye spot, and the plastid of the diatom endosymbiont [62, 63]. Transport of heme from the engulphed diatom for mitochondrial and cytosolic functions is obviously not possible. The same applies for the synthesis of tryptophan [64].

It is interesting enough to be mentioned here that it can also be a bacterial symbiont that becomes an active control agent of host sexual reproduction, as shown in the relationship between hymenopteran insects and the symbiotic bacteria, *Wolbachia*. These bacteria dramatically change the sex ratio of the insect host population in favor of females and force the insect to switch to parthenogenetic reproduction [65]. Such a change can be beneficial for the parasitic wasp because the asexual parthenogenetic insect is able to reproduce much faster than those that depend on sexual behavior.
